# Projecting Tree Species Composition Changes of European Forests for 2061–2090 Under RCP 4.5 and RCP 8.5 Scenarios

**DOI:** 10.3389/fpls.2018.01986

**Published:** 2019-01-11

**Authors:** Allan Buras, Annette Menzel

**Affiliations:** ^1^Professorship of Ecoclimatology, Technische Universität München, Freising, Germany; ^2^Land-Surface-Atmosphere-Interactions, Technische Universität München, Freising, Germany; ^3^Institute of Advanced Study, Technische Universität München, Garching, Germany

**Keywords:** tree-species vulnerability, climate-smart forests, forest-management adaptation, climate change, CMIP5 climate projections, climate analogs

## Abstract

Climate change poses certain threats to the World’s forests. That is, tree performance declines if species-specific, climatic thresholds are surpassed. Prominent climatic changes negatively affecting tree performance are mainly associated with so-called hotter droughts. In combination with biotic pathogens, hotter droughts cause a higher tree vulnerability and thus mortality. As a consequence, global forests are expected to undergo vast changes in the course of climate change. Changed climatic conditions may on the one hand locally result in more frequent dieback of a particular tree species but on the other hand allow other—locally yet absent species—to establish themselves, thereby potentially changing local tree-species diversity. Although several studies provide valuable insights into potential risks of prominent European tree species, we yet lack a comprehensive assessment on how and to which extent the composition of European forests may change. To overcome this research gap, we here project future tree-species compositions of European forests. We combine the concept of climate analogs with national forest inventory data to project the tree-species composition for the 26 most important European tree species at any given location in Europe for the period 2061–2090 and the two most relevant CMIP5 scenarios RCP 4.5 and RCP 8.5. Our results indicate significant changes in European forests species compositions. Species richness generally declined in the Mediterranean and Central European lowlands, while Scandinavian and Central European high-elevation forests were projected an increasing diversity. Moreover, 76% (RCP 4.5) and 80% (RCP 8.5) of the investigated locations indicated a decreasing abundance of the locally yet most abundant tree species while 74 and 68% were projected an increasing tree-species diversity. Altogether, our study confirms the expectation of European forests undergoing remarkable changes until the end of the 21st century (i.e., 2061–2090) and provides a scientific basement for climate change adaptation with important implications for forestry and nature conservation.

## Introduction

In the course of climate change, the World’s forests will likely undergo significant changes. This is because the frequency of heat waves likely will increase (IPCC, 2014) which in combination with long-lasting drought spells results in so called “global change type droughts,” “hotter droughts,” or compound events and eventually an increased tree mortality (Breshears et al., 2005; Allen et al., 2010, 2015; Martínez-Vilalta et al., 2012; Cailleret et al., 2017; Zscheischler and Seneviratne, 2017; Buras et al., 2018; Choat et al., 2018; Zscheischler et al., 2018). Consequently, tree species distributional ranges and the composition of forests will likely change (Pastor and Post, 1988; Hogg and Hurdle, 1995; Sykes and Prentice, 1996; Rigling et al., 2013; Fekete et al., 2017; Scherrer et al., 2017). Because of changing distributional tree species ranges and typically long rotation periods (often more than 100 years), forests have to be adapted to anticipated climate conditions by altering management strategies and selecting better adapted and therefore more resilient tree species (Halofsky et al., 2018; Sousa-Silva et al., 2018). In this context, foresters, stakeholders, and policy makers are targeting at so-called climate-smart forests (Halofsky et al., 2018; Nabuurs et al., 2018).

Already nowadays, the impact of hotter droughts on forests is visible (Breshears et al., 2005; Allen et al., 2010, 2015; Cailleret et al., 2017). Regarding European forests, the economically most important and spatially most abundant tree species of Scots pine (*Pinus sylvestris*, L.) and Norway spruce [*Picea abies*, (L.) Karst.] are regionally experiencing increased mortality rates (Bigler et al., 2006; Kohler et al., 2010; Lévesque et al., 2013; Rigling et al., 2013; Zang et al., 2014; Huang et al., 2017; Martínez-Sancho et al., 2017; Rehschuh et al., 2017; Buras et al., 2018). But also the widespread European beech (*Fagus sylvatica*, L.) is likely to suffer (Scharnweber et al., 2011, 2013; Dulamsuren et al., 2017; Huang et al., 2017; Walentowski et al., 2017) while other abundant species such as oak [*Quercus robur*, L. and *Quercus petraea*, (Matt.) Liebl.] may benefit from climate change (Scharnweber et al., 2011, 2013; Perkins et al., 2018).

Given their dominance within European forest ecosystems as well as their economic importance, it is crucial to evaluate the vulnerability of these tree species to anticipated climate change. Moreover, since some of these species are likely to decline under climate change, the key question arises, which other tree species feature a higher resilience to anticipated climate conditions and thus may be considered suitable alternatives (Walentowski et al., 2017). Several case studies have already provided valuable insights on potential reactions of selected species to projected climate (Sykes and Prentice, 1996; Dulamsuren et al., 2010, 2017; Scharnweber et al., 2011, 2013; Lévesque et al., 2013; Rigling et al., 2013; Zang et al., 2014; Rehschuh et al., 2017; Walentowski et al., 2017; Buras et al., 2018). However, these studies lean on different methodological approaches ranging from tree-ring analyses (Scharnweber et al., 2011, 2013; Lévesque et al., 2013; Zang et al., 2014; Dulamsuren et al., 2017; Rehschuh et al., 2017; Buras et al., 2018), over common-garden experiments also known as provenance trials (Martinez-Meier et al., 2008; Huang et al., 2017), to various types of species-distribution models (Sykes and Prentice, 1996; Hijmans and Graham, 2006; Walentowski et al., 2017) and are often restricted to a certain region and/or a relatively small number of tree species.

Another means to estimate the suitability of a given tree species under different climate conditions is related to the concept of climate analogs. Climate analogs have previously been used to assess climate-change induced ecosystem and land-use vulnerability and land-use change as well as to identify tree species or provenances which are more resilient to anticipated climate change (Hogg and Hurdle, 1995; Ford et al., 2010; Leibing et al., 2013; Luedeling et al., 2014). The idea of climate analogs is to identify regions in space and time which feature more or less similar climatological properties in comparison to a selected location at a given time. Since these regions feature similar climatological properties, they likely also provide suitable growing conditions for the same tree-species assemblages, as long as the considered climate parameters are relevant for tree ecophysiology. Identifying regions which currently feature climate conditions that are analogous to the projected conditions of a given site and determining the tree species which successfully survive under these “future analogs” thus may allow for depicting those tree species which are able to cope with anticipated climate conditions.

Under this framework, we here combine the concept of climate analogs with an ensemble of downscaled climate projections (CMIP5) and a recently established European forest inventory (Mauri et al., 2017) to project tree species composition changes of European forests under RCP 4.5 and RCP 8.5 scenarios until the end of the 21st century. By doing so, we seek to (1) provide detailed insights into the possible development of Europe’s most abundant tree species, (2) identify tree species which may potentially become more relevant for European forestry under anticipated conditions, and (3) outline the potential change in local tree-species abundance and composition which is linked to tree-species diversity. Thereby, we (4) aim at identifying potential hotspots of forest vulnerability which should be given special attention in the context of adapting European forests to climate change.

## Materials and Methods

### Data

#### Statistically Downscaled Climate Projections

For the climate analog computations, we made use of statistically downscaled CMIP5 climate projections. Considering 16 different models, we downloaded historical (1961–1990) and future (2061–2090) scenario projections of monthly minimum, mean, and maximum temperature as well as monthly precipitation sums. Regarding the future projections, we considered the RCP scenarios RCP 4.5 and RCP 8.5. Thus, altogether 192 datasets (4 variables × 16 models × 3 scenarios) were obtained, which were downloaded via various Earth System Grid Federation data nodes^1^ (for details see Supplementary Table S1). The selection of the particular 16 models as well as the particular period 2061–2090 was based on the availability of relevant climate projections covering a period of 30 years at the end of the 21st century at the ESGF data nodes. We are aware of that choosing only 16 CMIP5 models, may lead to a certain underrepresentation of projection spread (McSweeney and Jones, 2016). In this context, McSweeney and Jones (2016) recommended using at least 13 models to account for model uncertainty since they on average represent more than 80% of the overall spread of CMIP5 climate projections. Therefore, it seems likely that the selected 16 models represent model variability to a high degree.

Since the downloaded models had a rather coarse spatial resolution (in the order of 0.75–3°) and moreover different resolutions leading to differing overlaps of grid cells, we statistically downscaled all projections using Climate Research Unit gridded monthly minimum, mean, and maximum temperature [CRU TS version 4.01^2^ (Harris et al., 2014)] as well as Global Precipitation Climatology Center gridded monthly precipitation sums [GPCC^3^ (Rudolf and Ziese, 2011)] to a common 0.5° spatial resolution. Statistical downscaling was undertaken using the delta-method. That is, for the baseline period with temporal overlap between gridded and projected climate data (i.e., 1961–1990) we for each projected variable (CMIP5) and grid cell computed its mean monthly climatological difference to the corresponding gridded variable (CRU and GPCC, respectively). Thereby, we for each variable obtained a grid at 0.5° spatial resolution, representing the difference between the 1961–1990 monthly climatology of projected and gridded climate variables. In case of uneven spatial overlap of the two different grids, we computed the corresponding zonal mean. These climatological differences were subsequently added to the CMIP5 climate projections (both historic and future scenarios) to finally obtain climate projections at 0.5° spatial resolution and referenced to the baseline period 1961–1990. Due to this downscaling procedure, the temperature and precipitation climatology of all historic projections matched exactly those of the gridded data within the baseline period. However, the temporal patterns varied among models in dependence of particular model specifications which cause the typically observed spread of model projections (McSweeney and Jones, 2016).

Statistically downscaled projections were further used to compute ecologically meaningful variables related to growing season temperatures, length of the growing season, climatic water balance, and seasonality (Metzger et al., 2013). Computation of such generally more meaningful variables with regard to plant ecology is needed to better represent the actual “climate envelopes” of tree species (Hijmans and Graham, 2006). In order to simplify the multidimensionality of the obtained data, we computed the spatiotemporal mean for each of the obtained variables (i.e., for each grid cell and time step) across the 16 selected models. To select the most appropriate variables for the definition of climate analogs, we finally tested a large number of various variable combinations regarding their ability to represent the well-known Köppen-Geiger climate classification (Köppen, 1900; Geiger, 1961) as well as two more recent global climate classifications (Olson et al., 2001; Metzger et al., 2013). This was done by comparing corresponding classifications representative of the respective variable selection (computed to comprise 32 climate zones using the partitioning around medoids method) with the considered, existing classifications (i.e., Köppen-Geiger, Metzger, and Olson). As a measure of match, we computed the so-called Kappa-statistic and used the threshold values introduced by Monserud and Leemans (1992) which revealed good to very good agreement (Kappa ranging from 0.58 to 0.79) of the finally selected variable combination with the chosen classifications. Following this approach, we eventually selected 11 variables which are listed and described in detail in Supplementary Table S2. The corresponding climate classification is shown in the supplementary (Supplementary Figure S1). The spatial extent of the generated climate parameters was finally matched with the forest inventory data (see Section “Forest Inventory Data”), i.e., longitude ranging from 10.75° W to 32.75° E and latitude ranging from 35.75° N to 70.75° N resulting in altogether 3,949 considered grid cells.

#### Forest Inventory Data

As a baseline for current tree species distributions across Europe, we downloaded the recently published EU-Forest dataset (Mauri et al., 2017). This dataset contains altogether 588,983 tree-species records (for details see Mauri et al., 2017). Due to low spatial and climatological representativity we did not consider data from the Canary Islands, finally resulting in 586,862 records representing altogether 242 different tree species distributed across Europe. Since many of these species were only represented at very few locations and thus would not allow for a meaningful representation of their current potential distribution, we decided to only include the 26 species which each at least represented 1% of the total dataset. By doing so, we eventually considered 82% of the EU-Forest dataset for our analyses (for an overview on the selected species see Supplementary Table S3).

### Data Processing and Statistical Analyses

#### Computation of Climate Analogs

To map recent and potential future species distributions we computed climate analogs and merged those with the EU-forest dataset (see Section “Mapping Current and Future Tree-Species Distributions”). Here, we used climate analogs to identify regions which currently feature climate conditions that are more or less similar to the projected conditions of a given site. Depending on the selected target projection we in the following call these climates *current* analogs if considering current climates that are analogous to the current climate of a given site vs. *future* analogs if considering current climates that are analogous to the projected future climate of a given site.

Prior to defining current and future analogs, we standardized (z-transformed) each of the selected 11 variables separately. For the standardization of historic and future projections we used the mean and SD of the historic baseline period (1961–1990). Thereby, we for each variable obtained records representing unitless SDs from the variable-specific historic mean. Doing so allowed for equally weighing the variables while retaining the offset of projected future climates in relation to the historic baseline period.

To define current/future climate analogs, we then computed the Manhattan distance (Black, 2006) between the standardized current/future climate variables of a given site and the historic climate variables of all considered grid cells. Consequently, each of the considered grid cells obtained a value which resembled the climatic distance (considering all 11 variables) of that grid cell to the selected site and projection. That is, the lower this climatic distance, the more similar the climate of the respective grid cell to the projected current/future climate of the selected site.

Finally, we had to define a threshold of climatic distance below which a climate is considered analogous to the climate at a location of interest. For reasons of objectivity and reproducibility we based this threshold on the average first percentile of current climate distances computed over all grid cells. That is, we for each of the 3,949 grid cells computed its climatic distance to the current climate of the remaining 3,948 grid cells and extracted the first percentile of the grid-cell specific climate distances. The mean over all 3,949 first percentiles of grid-cell specific climate distances we took as an objective and uniform measure for defining climate analogs. A verification of the first percentile was performed manually by computing 100 different climate analogs and visually comparing the climate diagrams (Walter and Lieth, 1960) of analogous grid cells with those of the selected site. Since climate diagrams in most cases matched very well, we considered the selected threshold of the first percentile as suitable. We also assessed other percentiles, e.g., 5 permille and the second percentile but thereby we either obtained too small climate analog regions for several locations (5 permille) or included areas, whose extreme climate diagrams did not match well within the analogous region (second percentile). The match between actual and projected historic species distributions may be considered a further validation procedure (see Section “Mapping Current and Future Tree-Species Distributions”).

#### Mapping Current and Future Tree-Species Distributions

The EU-forest dataset was used as the basis for mapping current and future tree-species distributions. First of all, we had to transform the point-based records into a gridded format that matched the resolution and extent of computed climate analogs (see Section “Computation of Climate Analogs”). Therefore, we computed the relative abundance of a given species within each grid cell. For this, all EU-forest records within a specific grid cell were pooled by species and the relative contribution of each species computed, thereby obtaining values between 0 and 1. That is, if a species had no records within that grid cell it would obtain the value 0, whereas if a species was the only species occurring within that cell it would obtain the value 1, while if a species would co-occur with other species it would obtain a value between 0 and 1 reflecting its proportional contribution to grid-cell specific tree species abundances.

**FIGURE 1:**
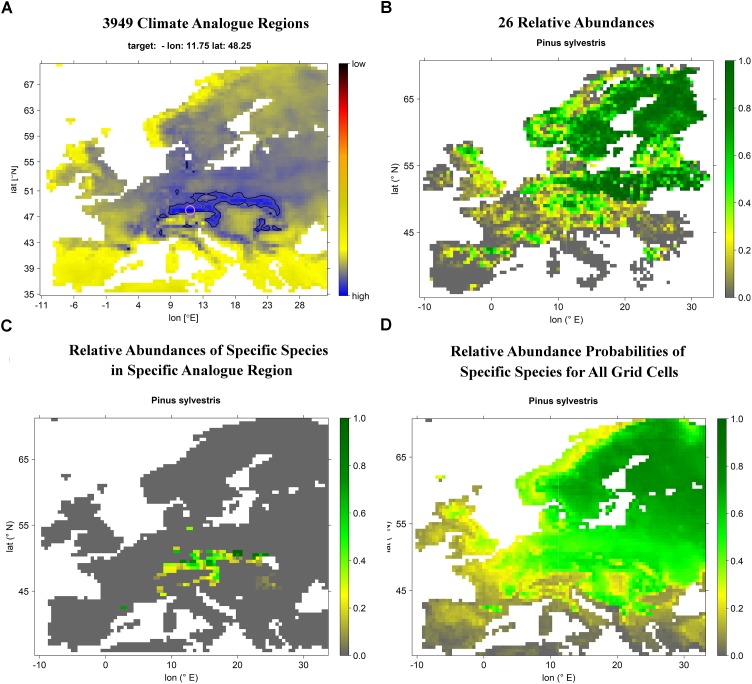
Schematic flow chart depicting the processing of climate projections and EU-forest inventory data to eventually obtain projected species distribution maps (here using the grid cell corresponding with the location of Munich and Scots pine projected for current climate conditions as an example). **(A)** For each of the 3,949 grid cells current and future analogs are computed resulting in 3,949 different climate analog regions. **(B)** For each species, relative abundance per grid cell is computed, resulting in 26 species-specific relative abundance maps. **(C)** Intersection of the current/future climate analog region with the species-specific relative abundance map allows for computing the mean relative abundance of a given species within the corresponding climate analog region which is taken as local relative abundance probability of the corresponding grid cell. **(D)** Computing local relative abundance probabilities for each of the 3,949 grid cells allows for projecting the relative abundance probability of a specific species under a certain scenario across Europe. Please note the comparably good match between actual Scots pine relative abundance **(B)** and projected current Scots pine relative abundance probability **(D)**.

In the next step, these relative abundance maps were merged with the climate analogs. Thereby, we obtained the occurrence probability of a given species at a specific site with a certain climate projection (historic or future). For this, we first computed the according site- and projection-specific (historic or future) climate analog and secondly averaged the relative abundances of the corresponding analog region per species. That is, if a species expressed fairly high (low) abundance in most of the analog grid cells, it would also gather a high (low) average abundance for the respective site and projection. This procedure was carried out for each of the 26 selected species and applied to each of the 3,949 grid cells, to finally obtain European-wide occurrence probabilities of the 26 most abundant tree species under current and future climate projections. Figure 1 depicts a schematic flow-chart of our methodological approach.

To assess how well the projected relative abundance probabilities represented observed relative abundances as obtained from EU-forest, we for each of the 26 species computed Spearman’s rank correlations between observed and projected current relative abundance. Correlation scores from 0.43 to 0.76 (mean 0.58) indicated moderate to good agreement between observed and projected relative abundance. Observed and projected current relative abundance maps are shown along with the species-specific correlations between observations and projections in the supplementary information (Supplementary Figures S2–S8).

#### Evaluation

To visualize the change of European forests tree species composition (see Section “Mapping Current and Future Tree-Species Distributions”) we first of all plotted current and future relative abundance probabilities for the four currently most abundant European tree species, i.e., *P. sylvestris* (Scots pine), *P. abies* (Norway spruce), *F. sylvatica* (European beech), and *Q. robur* (English oak). In combination, these four species make up 44% of the considered forest inventory data. As a proxy for tree-species relevance for forestry within the different scenarios, we computed the mean relative abundance probability for each species across all grid cells for current and future conditions. That is, species with high mean relative abundance probabilities in a given scenario feature a high relevance for forestry and *vice versa*. As a measure of change for the general appearance of forests, we moreover computed the relative change of the currently most dominant tree species for each of the 3,949 grid cells. That is, for each grid cell the currently dominant tree species was determined and its relative abundance probability of the two future scenarios for the same grid cell was put in relation to its current relative abundance probability. Finally, as a measure of tree-species diversity changes, we for each grid cell computed Shannon’s H (Shannon, 1948) and expressed the absolute change (i.e., the difference) of the two future scenarios in relation to the current value. All analyses were performed in, R’ (version 3.3.3., R Core Team, 2017) extended for the packages SPEI (Beguería and Vicente-Serrano, 2013) and lattice (Sarkar, 2008).

## Results

**FIGURE 2:**
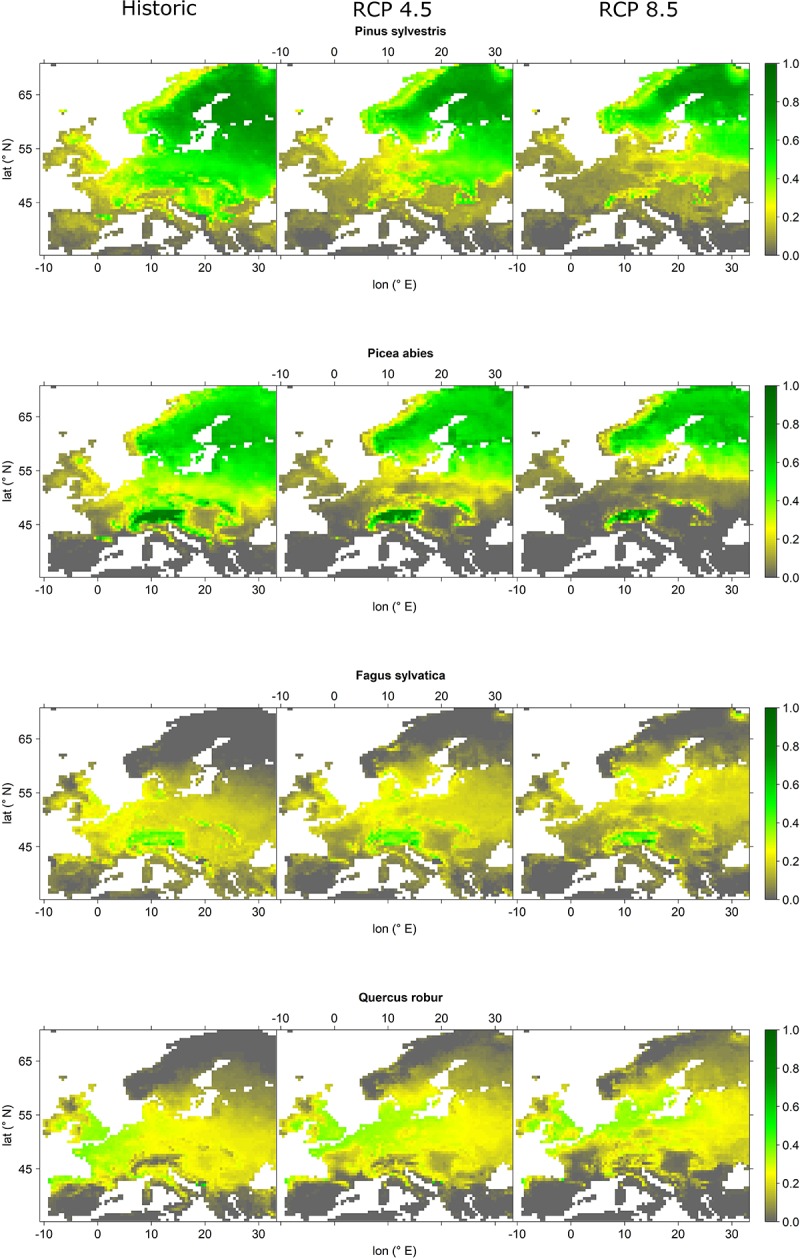
Projected relative abundance probabilities for the four most abundant tree species *Pinus sylvestris*, *Picea abies*, *Fagus sylvatica*, and *Quercus robur* for current analogs (left panels), RCP 4.5 analogs (mid panels), and RCP 8.5 analog (right panels). Relative abundance probability increases from gray over yellow to green colors.

**FIGURE 3:**
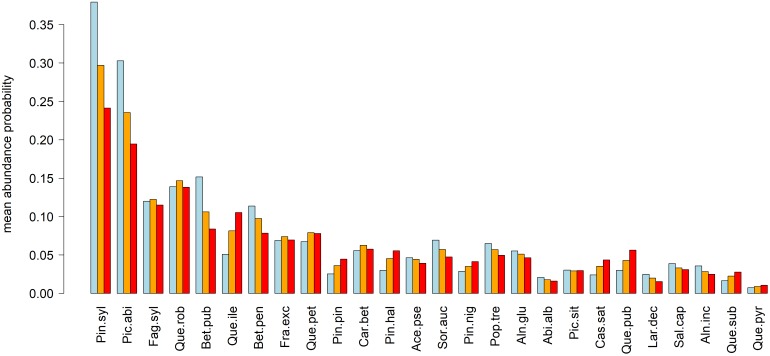
Mean relative abundance probabilities under current (blue) and projected future (orange = RCP 4.5, red = RCP 8.5) climate conditions. Species abbreviations: Pin.syl, *Pinus sylvestris*; Pic.abi, *Picea abies*; Fag.syl, *Fagus sylvatica*; Que.rob, *Quercus robur*; Bet.pub, *Betula pubescens*; Que.ile, *Quercus ilex*; Bet.pen, *Betula pendula*; Fra.exc, *Fraxinus excelsior*; Que.pet, *Quercus petraea*; Pin.pin, *Pinus pinaster*; Car.bet, *Carpinus betulus*; Pin.hal, *Pinus halepensis*; Ace.pse, *Acer pseudoplatanus*; Sor.auc, *Sorbus aucuparia*; Pin.nig, *Pinus nigra*; Pop.tre, *Populus tremula*; Aln.glu, *Alnus glutinosa*; Abi.alb, *Abies alba*; Pic.sit, *Picea sitchensis*; Cas.sat, *Castanea sativa*; Que.pub, *Quercus pubescens*; Lar.dec, *Larix decidua*; Sal.cap, *Salix caprea*; Aln.inc, *Alnus incana*; Que.sub, *Quercus suber*; Que.pyr, *Quercus pyrenaica*.

The four most abundant tree species indicated prominent changes in relative abundance probabilities, particularly for RCP 8.5 projections (Figures 2, 3). All of the four species indicated a declining abundance probability at southern latitudes and lower elevations. Based on declining abundance probabilities, Scots pine indicated a retreat from Central and Southeast Europe to the higher elevations of the Alps and the Carpathians, as well as Northern Europe. The same was observed for Norway spruce. However, for this species the retreat appeared even more pronounced, i.e., under RCP 8.5 projections it completely disappeared from Central European lowlands with last refugia in the Alps and the Carpathians as well as in Scandinavia north of 60° latitude. European beech also indicated a northward migration, i.e., abundance probabilities significantly decreased over large parts of Central Europe but increased in Southern Scandinavia. Finally, English oak retreated from its southernmost locations such as large parts of France and the Pannonian Basin and increased its abundance in Southern Scandinavia.

While Scots pine and Norway spruce expressed a decrease in the order of 5 (RCP 4.5) to 15% (RCP 8.5) of mean relative abundance probability across Europe, European beech and English oak retained their mean relative abundance probability for both scenarios (Figure 3). Despite possible losses in abundance probability, these four tree-species remained the most abundant across Europe. Tree species with remarkable increases (2–6%) of abundance probabilities were *Quercus ilex*, *Pinus pinaster*, *Pinus halepensis*, *Pinus nigra*, *Castanea sativa*, and *Quercus pubescens*, all of which seemed to expand their current distributional range from the Mediterranean to Central Europe. In contrast, declining species mostly featured a northward migration of their distributional range rather than an expansion (Figure 3 and Supplementary Figures S9–S14).

**FIGURE 4:**
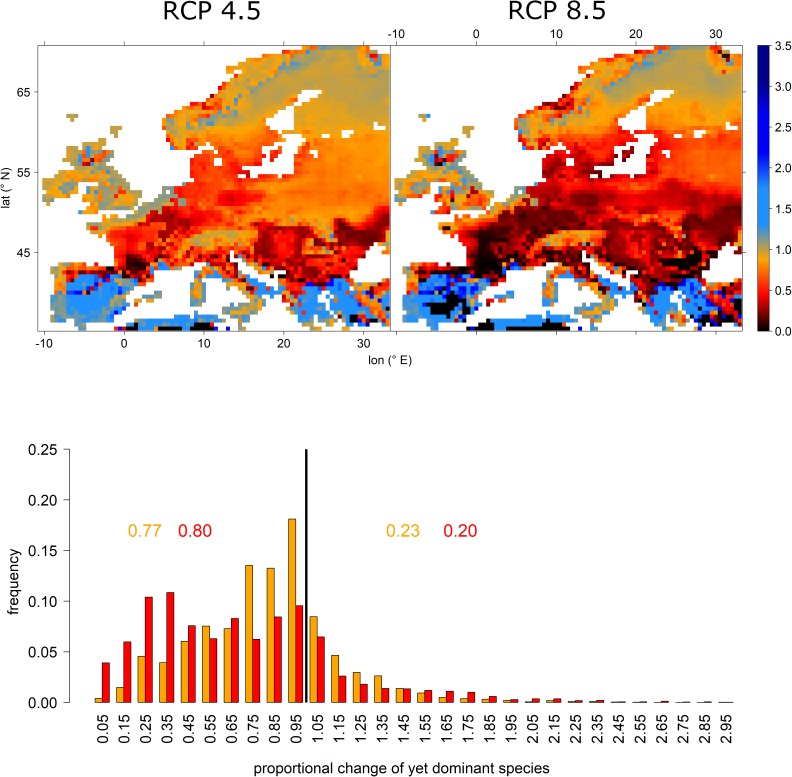
Proportional change of relative abundance probability of the yet dominant tree species per grid cell for RCP 4.5 (upper left) and RCP 8.5 (upper right) and a histogram depicting the frequency of features shown in the upper panels. Upper panels: black, red, and orange colors indicate losses (i.e., proportions below 1), while blue colors indicate an increasing abundance. The lower panel depicts the frequencies of relative abundance probability changes of the yet dominant tree species. Here, orange colors refer to RCP 4.5 projections, while red colors indicate RCP 8.5. The black vertical line indicates the threshold between loss and gain. The inserted values refer to the cumulative frequencies of losses (left of black vertical line) and gains (right of black vertical line) for RCP 4.5 (orange) and RCP 8.5 (red).

Projected relative abundance changes of currently dominant tree species depicted large losses for most of Europe. However, large areas in the Mediterranean as well as some higher elevations in England and Scandinavia expressed increasing abundances. That is, for many European regions, projections indicated the currently most abundant tree species to experience a significant decline in abundance (Figure 4 upper panel). More specifically, under RCP 4.5 77% of all grid cells expressed losses which increased to 80% under RCP 8.5. However, under the more extreme RCP 8.5 scenario, many more grid cells expressed remarkable losses, i.e., for more than 30% of grid cells less than 50% of current relative abundance of the dominant species were retained (Figure 4 lower panel).

**FIGURE 5:**
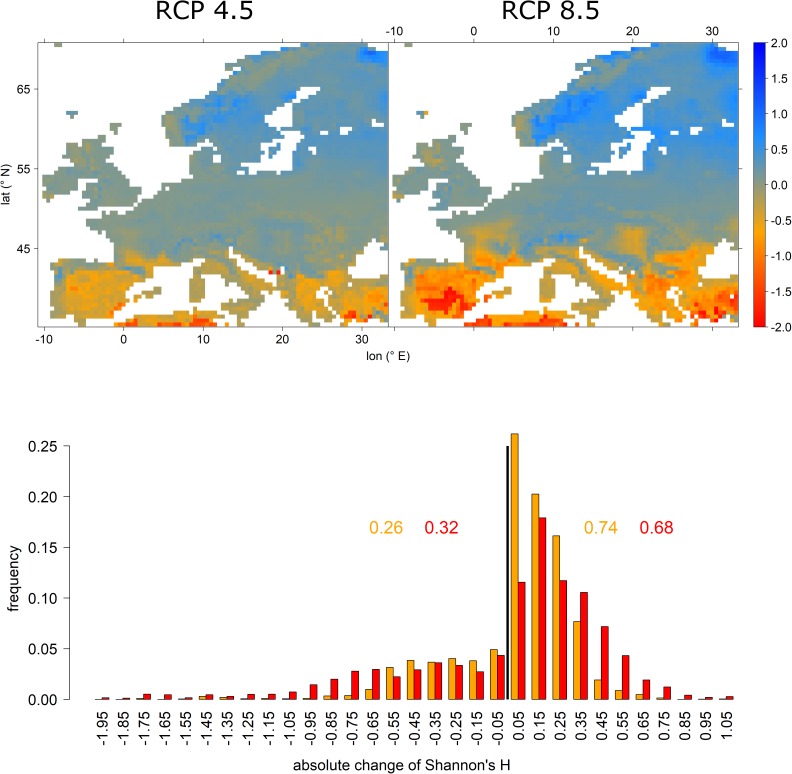
Absolute change in Shannon’s H for RCP 4.5 (upper left) and RCP 8.5 (upper right) and a histogram depicting the frequency of features shown in the upper panels. Upper panels: red and orange colors indicate a loss in tree-species diversity while blue colors demarcate increasing tree-species diversity. The lower panel depicts the frequencies of absolute change of Shannon’s H, i.e., tree-species diversity. Here, orange colors refer to RCP 4.5 projections, while red colors indicate RCP 8.5. The black vertical line indicates the threshold between loss and gain. The inserted values refer to the cumulative frequencies of losses (left of black vertical line) and gains (right of black vertical line) for RCP 4.5 (orange) and RCP 8.5 (red).

Shannon’s H expressed features complementary to relative abundance changes (Figure 5 vs. Figure 4). That is, while tree-species diversity decreased in the Mediterranean, it generally increased in Central and Northern Europe, thereby mirroring the immigration of new species into those regions (Figure 5 upper panels). Under RCP 4.5 74% of grid cells expressed increasing tree-species diversity while it was only 68% under RCP 8.5 (Figure 5 lower panel). However, under RCP 8.5 the distribution was shifted toward higher increases in tree-species diversity, particularly in Scandinavia.

## Discussion

### The Projected Face of European Forests

Based on our projections, it seems likely that the appearance of European forests will change remarkably until the end of the 21st century. Most prominent changes are related to the northward migration of the four most abundant species, i.e., Scots pine, Norway spruce, European beech, and English oak and at the same a remarkable decrease in abundance in Central Europe, particularly regarding Norway spruce and Scots pine (Figure 2). Other currently abundant species such as *Betula pubescens*, *Betula pendula*, *Q. petraea*, and *Sorbus aucuparia* featured similar projections (Figure 3 and Supplementary Figures S9–S11). Considering whole Europe, the relative importance of Scots pine and Norway spruce—the currently economically most important tree species—decreased, while for European beech and English oak, the relative importance remained unchanged (Figure 3). Tree species that were projected to partly fill the resulting gaps were *Q. ilex*, *P. pinaster*, *P. halepensis*, *P. nigra*, *C. sativa*, and *Q. pubescens* (Figure 3 and Supplementary Figures S9–S14). For large parts of Central and Northern Europe, the currently dominant tree species experienced remarkable reductions in abundance, particularly for RCP 8.5 while for most Mediterranean areas an increase in abundance of the currently dominant species was projected (Figure 4). The projected migrations and changes in abundance resulted in an increasing tree-species diversity in most regions north of the Alps and a decreasing tree-species diversity in the Mediterranean (Figure 5).

In combination, these results outline potential hotspots of vulnerability, particularly in Southern France, Spain, and on the Italian peninsula due to both decreasing frequencies of currently most dominant tree species as well as decreasing tree-species diversity even under the less extreme RCP 4.5 scenario (Figures 4, 5). Other potential hotspots of forest vulnerability—though to a lower degree—are located in Bulgaria and Romania as well as the Pannonian Basin, particularly under the RCP 8.5 scenario. For these hotspots, it according to our projections seems likely that the currently most abundant tree species will experience a remarkable decline in abundance, while at the same, species diversity will decrease. Consequently, these regions should be given special attention in the context of adapting local forests to anticipated climate change and tree-species should be selected carefully since the species-portfolio of those regions is likely to decline.

### Species Projections in the Scientific Context

The projected changes of the four most abundant species are in line with recent research. First of all, for Scots pine an increasing mortality at its southern distributional margin was observed over the last decades, e.g., on the Iberian peninsula after a severe drought in 2005 (Galiano et al., 2010, 2011), in inner-alpine dry valleys, for instance in 1998 and 2003 (Rebetez and Dobbertin, 2004; Bigler et al., 2006; Pichler and Oberhuber, 2007), and recently also Central European lowlands after the hot and dry summer of 2015 (Buras et al., 2018). Secondly, Norway spruce is well-known for its high drought-susceptibility (Kohler et al., 2010; Lévesque et al., 2013; Zang et al., 2014; Rehschuh et al., 2017). Since the frequency and intensity of drought spells are projected to increase in Central Europe (Horton et al., 2015; Pfleiderer and Coumou, 2018), the disappearance of Norway spruce from Central European lowlands in our projections seems reasonable. Finally, our projections suggested beech to express a more pronounced decline in Central Europe compared to English oak, although both species were relatively less affected in comparison to Scots pine and Norway spruce. On the one hand, this is in line with Zang et al. (2014), who reported a lower drought susceptibility of beech in comparison to Norway spruce. On the other hand, the higher drought susceptibility of beech in comparison to English oak has been reported on the basis of dendro-ecological investigations (Scharnweber et al., 2011, 2013; Walentowski et al., 2017) and species distribution models (Walentowski et al., 2017).

Considering less abundant species, the projected northward expansion of *Q. ilex* also finds support in the literature. Based on tree-ring analyses, *Q. ilex* was shown to decline under increasing water stress, but to benefit from increasing winter temperatures (Gea-Izquierdo et al., 2011), which is in line with the projected northward expansion and decline in the driest regions in Spain (Supplementary Figure S9). Also, the increasing importance of *Q. pubescens* in Central European lowlands (Supplementary Figure S13), finds support in the literature. That is, pubescent oak has been reported to potentially replace Scots pine in dry inner-alpine valleys due to a comparably better adaptation to dry conditions (Eilmann et al., 2009; Rigling et al., 2013). The projected northward migration of *P. nigra* (Supplementary Figure S11) is supported by studies which reported a relatively high drought-susceptibility of black pine and increasing trends in needle defoliation in the Mediterranean which reflects its sensitivity to increasing temperatures (Linares and Tíscar, 2010; Carnicer et al., 2011). Moreover, its cold-hardiness was reported to be in the range of Central European species which renders it suitable for the projected northward migration (Kreyling et al., 2012). Also for *P. pinaster* and *P. halepensis* an increased leaf defoliation has been observed, indicating a declining performance of those species in the Mediterranean (Carnicer et al., 2011) which is also reflected in their projected northward migration in our study (Supplementary Figure S10). In conclusion, several of the presented projected changes of European tree-species distributions are supported by independent studies.

### Constraints and Limitations

Despite the support by independent studies, the methodological approach behind the presented projected tree-species distributions features certain constraints and limitations. Most of all, the rather coarse resolution of 0.5° yet needs further improvement. This is because at such coarse resolution, elevational differences are only poorly resolved—particularly in the inner-alpine valleys but also in other mountain areas such as the Norwegian fjords. Therefore, projected relative abundances of these regions have to be interpreted carefully. For instance, while it seems likely that Norway spruce and Scots pine refugia will remain at higher elevations in the Alps, these species will likely disappear from the dry inner alpine-valleys. To overcome these limitations, future studies should consider integration of higher resolved climate projections such as the EURO-CORDEX ensembles with 11 km spatial resolution (Jacob et al., 2014) or the WORLDCLIM projections with 1 km spatial resolution (Fick and Hijmans, 2017). However, since we wanted to account for model uncertainty by incorporating a comparably large number of different simulations and moreover encountered computational limitations, we here opted for downscaled projections derived from 16 different models. But even if increasing the spatial resolution to 1 km, unresolved sub-scale processed would remain. A focal point in the context of sub-scale processes is the forest edge, which recently was shown to feature a higher drought-induced mortality of Scots pine within the first 50 m of forest-edge distance (Buras et al., 2018) that may also hold true for other tree species. However, given the specific micro-climatic conditions at the forest edge (Chen et al., 1993, 1999), incorporation of these effects into projections of tree-species distributions that rely on gridded climate projections only remains a demanding challenge. Consequently, the forest edge needs to be given further attention in the context of climate-change induced tree vulnerability.

Another shortcoming of the presented approach is related to the current relative abundance of species. For instance, *Picea sitchensis* is yet mainly distributed in the United Kingdom and Denmark, which may introduce certain biases regarding its projected distribution. That is, although it also is capable of growing under more continental conditions, the high abundance in the observational data mainly restricts it to coastal climates in the projected relative abundances. This is also the main reason, why we focused our evaluation on the four most abundant tree species. However, to also include species with projected increasing relative abundances, we decided to consider all species which cover at least 1% of the EU-forest data. Another constraint in this context is related to other theoretically important tree species that were not considered in our approach due to a marginal representation in the EU-forest data. For instance, Walentowski et al. (2017) reported *Acer campestre*, *Sorbus torminalis*, *Sorbus aria*, *Ulmus minor*, and *Tilia platyphyllos* to be well adapted to anticipated climate conditions, all of which were not considered here due to low representation. Within this context it moreover is important to mention, that based on the utilized forest-inventory data, we were not able to incorporate tree species from the Mediterranean coast of North Africa, which may qualify as potential alternative tree species for the European Mediterranean under projected climate conditions. Therefore, the decline in tree-species diversity for Mediterranean regions has to be interpreted in the context of the underlying data. That is, the depicted declining tree-species diversity in those regions highlights a substantial loss in diversity regarding Europe’s 26 currently most abundant tree species, and thus that local foresters should consider non-native tree species from outside of Europe as potential alternatives.

The rigorous definition of the climate analog threshold to the average first percentile over all European grid cells also has certain caveats. Although the validation assessments confirmed this threshold superior in comparison to others, some likely errors resulted as for instance the projected increase in relative abundance of beech and oak on the Kola Peninsula (Figure 2). One way to cope with this, is to incorporate additional climate parameters into the computation of climate distances. However, in the extensive trials undertaken to identify the best-suited variable combination, we eventually decided for the current selection, which may be considered the best compromise among available variables, particularly since the match between actual and projected current tree-species distributions were fairly good (see Section “Mapping Current and Future Tree-Species Distributions” and Supplementary Figures S2–S8).

Our projections of future tree-species distributions were restricted to the RCP 4.5 and RCP 8.5 scenarios. However, this does not represent the full range of available climate scenarios for the end of the 21st century. For instance, the Paris agreement obtained at the conference of the parties 21 aimed at limiting global warming to well below 2°C and ideally 1.5°C (Schleussner et al., 2016). However, the scenarios RCP 4.5 and RCP 8.5 on average result in a global warming of 2.4 and 4.1°C, respectively. Although this is higher than the aim of the Paris agreement, these projections appear to be realistic since it based on the current nationally stated mitigation ambitions seems likely that global temperatures will increase by 3°C until the end of the 21st century (IPCC, 2018).

Finally, our approach mainly relies on climatic properties of analog regions, while pathogens such as fungi and bark beetles were not considered. Although the risks associated with potential pathogens to some degree are reflected in current species abundances, other specific diseases are not included. On the one hand increasing drought frequency is considered an inciting factor for specific fungi and bark beetles that indirectly determine tree-species distributions via mortality (Bigler et al., 2006; Rehschuh et al., 2017; Buras et al., 2018) and likely are incorporated in our climate-based approach. But on the other hand, specific diseases or beetles which are only indirectly related to climate such as the Asian longhorn beetle or the fungi responsible for the remarkable ash die-back in Europe are likely not covered by our approach (MacLeod et al., 2002; Kirisits and Freinschlag, 2012).

Despite these limitations, the presented projections of relative species abundances may be considered a valuable estimate of anticipated forest change. Outlining the most prominent changes of tree-species distributions and identifying associated hotspots provides stake-holders, foresters, and ecologists with valuable insights into upcoming changes of forest compositions. However, given their currently relatively low abundance, the performance of some of the identified alternative species under climate change (such as *C. sativa*) should be investigated in more detail.

## Conclusion and Outlook

Our projections of tree-species distributions highlight prominent changes for Europe’s currently most abundant tree species. That is, based on our projections and due to increasing risks of drought-induced mortality Scots pine and Norway spruce will likely become unprofitable and consequently significantly decrease their abundance in Central European lowland forests in course of the anticipated climate change. Depending on the magnitude of climate change (RCP 4.5 vs. RCP 8.5), European beech and English oak may sustain in large parts of Central Europe. Potential alternative tree species identified here, such as *Q. ilex*, *P. nigra*, *P. halepensis*, *P. pinaster*, and *C. sativa* may be considered a meaningful replacement of locally declining tree species. However, other species which were not integrated into our approach because of low representation in the EU-forest dataset and were shown to be potential replacement species in other studies should also be taken into consideration. Moreover, our study identified hotspots of forest vulnerability in South France, Spain, Italy, the Pannonian Basin, Bulgaria, and Romania. Particularly in those areas, foresters, stakeholders, and nature conservationists should pay special attention to adapt local forests to anticipated changes well in time by selecting tree species that are adapted to anticipated climate conditions. To further refine our picture about future forests, subsequent studies should aim at incorporating higher resolved gridded climate projections into climate analog computations and extent the forest-inventory beyond Europe.

## Data Availability Statement

Upon publication of the manuscript, the datasets generated for this study can be found in the Github repository of AB. The R-code used for computation of climate analogs will soon be released as an “R”-package entitled “ClimCong.”

## Author Contributions

AB and AM designed the study. AB performed all statistical analyses and wrote the manuscript with valuable contributions by AM.

## Conflict of Interest Statement

The authors declare that the research was conducted in the absence of any commercial or financial relationships that could be construed as a potential conflict of interest.

## Footnotes

1 https://esgf.llnl.gov/

2 https://crudata.uea.ac.uk/cru/data/hrg/

3 https://www.esrl.noaa.gov/psd/data/gridded/data.gpcc.html

## Abbreviations

CMIP 5: Coupled Model Intercomparison Project Phase 5
RCP: relative concentration pathway
