# Supplementary material for: Projecting Tree Species Composition Changes of European Forests for 2061–2090 Under RCP 4.5 and RCP 8.5 Scenarios
Source: Front Plant Sci. 2019 Jan 11;9:1986. doi: 10.3389/fpls.2018.01986 (PMC6337730; doi:10.3389/fpls.2018.01986)
Supplement: Supplementary file 1 [file Table_1.DOCX]

Supplementary Material

**Projecting Tree Species Composition Changes of European Forests for 2061-2090 under RCP 4.5 and RCP 8.5 Scenarios**

**Allan Buras^1,2*^, Annette Menzel^1,3^**

*** Correspondence:** Dr. Allan Buras: allan@buras.eu

# Supplementary Figures and Tables

## Supplementary Figures


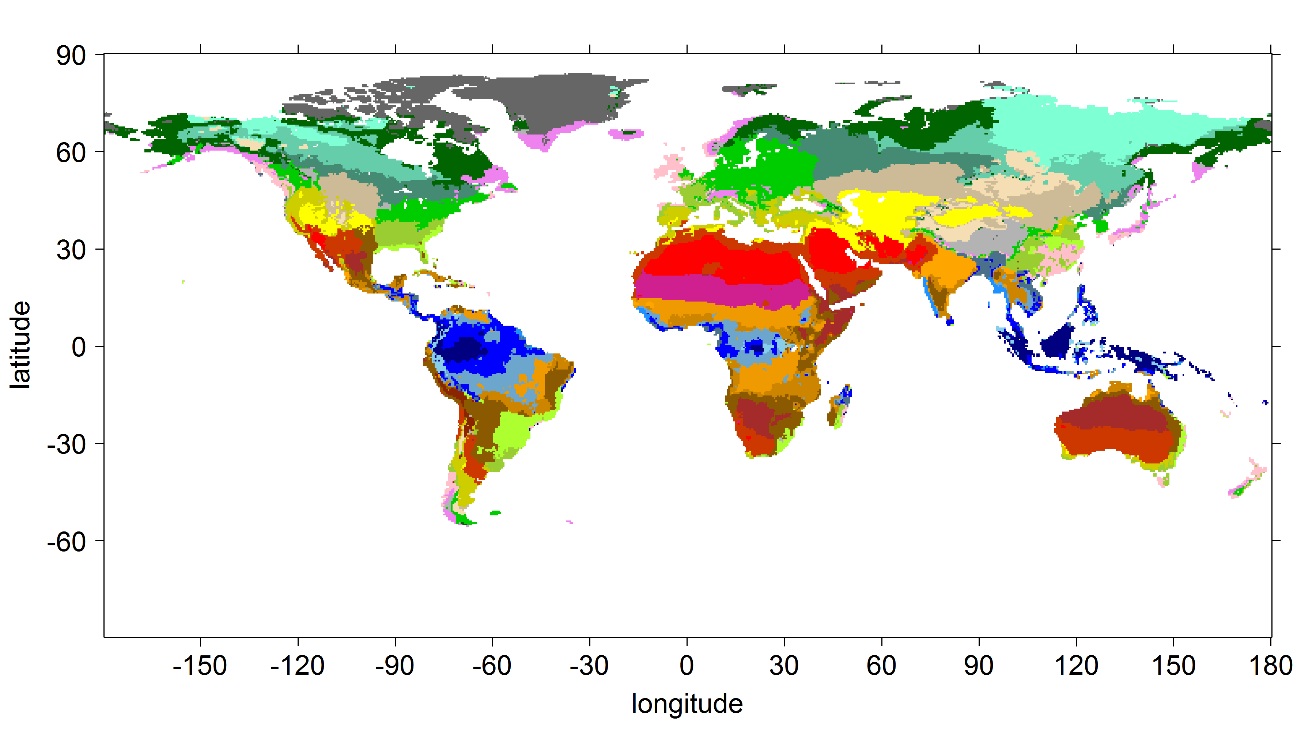


**Supplementary Figure S1.** Climate classification based on the 11 selected variables depicted in Table S2. The different colors refer to the 32 climate classes computed using a partitioning around medoids algorithm. These classes were used to assess the agreement with existing climate classifications (for details please see section 2.1.1).


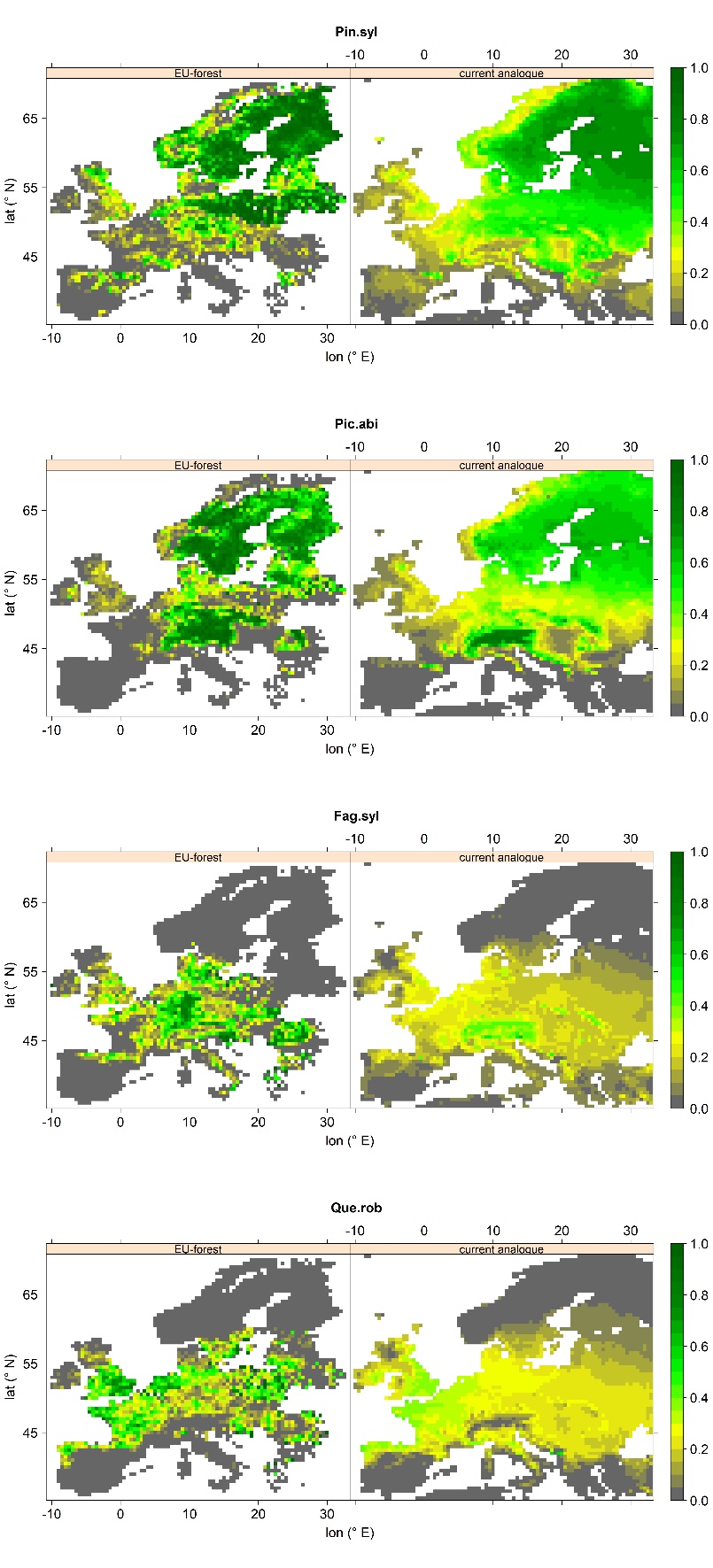


**Supplementary Figure S2.** Relative abundance of *Pinus sylvestris*, *Picea abies*, *Fagus sylvatica*, and *Quercus robur* according to EU-forest observations (left) and historic projections (right). Spearman’s rank correlation between EU-forest observations and historic projections are 0.76, 0.76, 0.71, and 0.73, respectively.


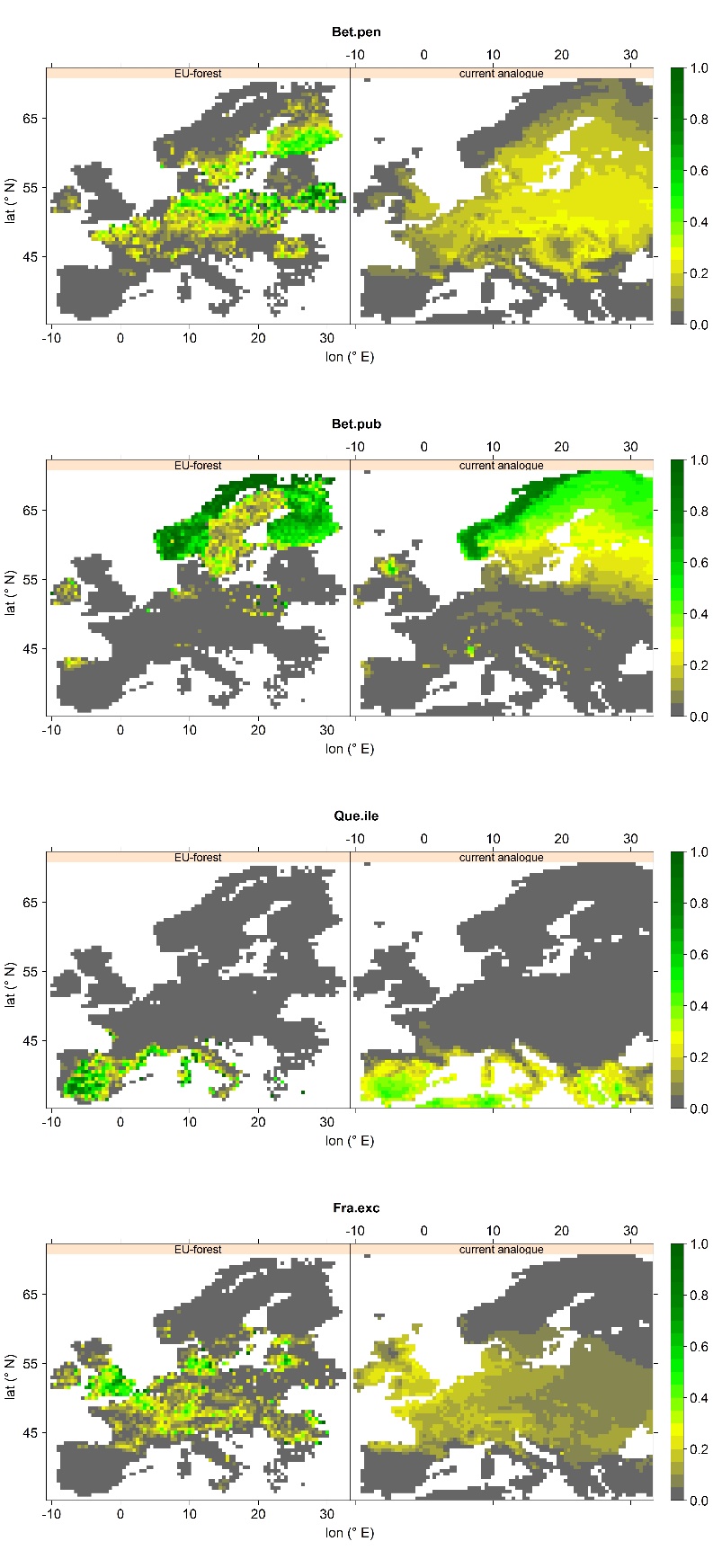


**Supplementary Figure S3.** Relative abundance of *Betula pendula*, *Betula pubescens*, *Quercus ilex*, and *Fraxinus excelsior* according to EU-forest observations (left) and historic projections (right). Spearman’s rank correlation between EU-forest observations and historic projections are 0.63, 0.73, 0.55, and 0.66, respectively.


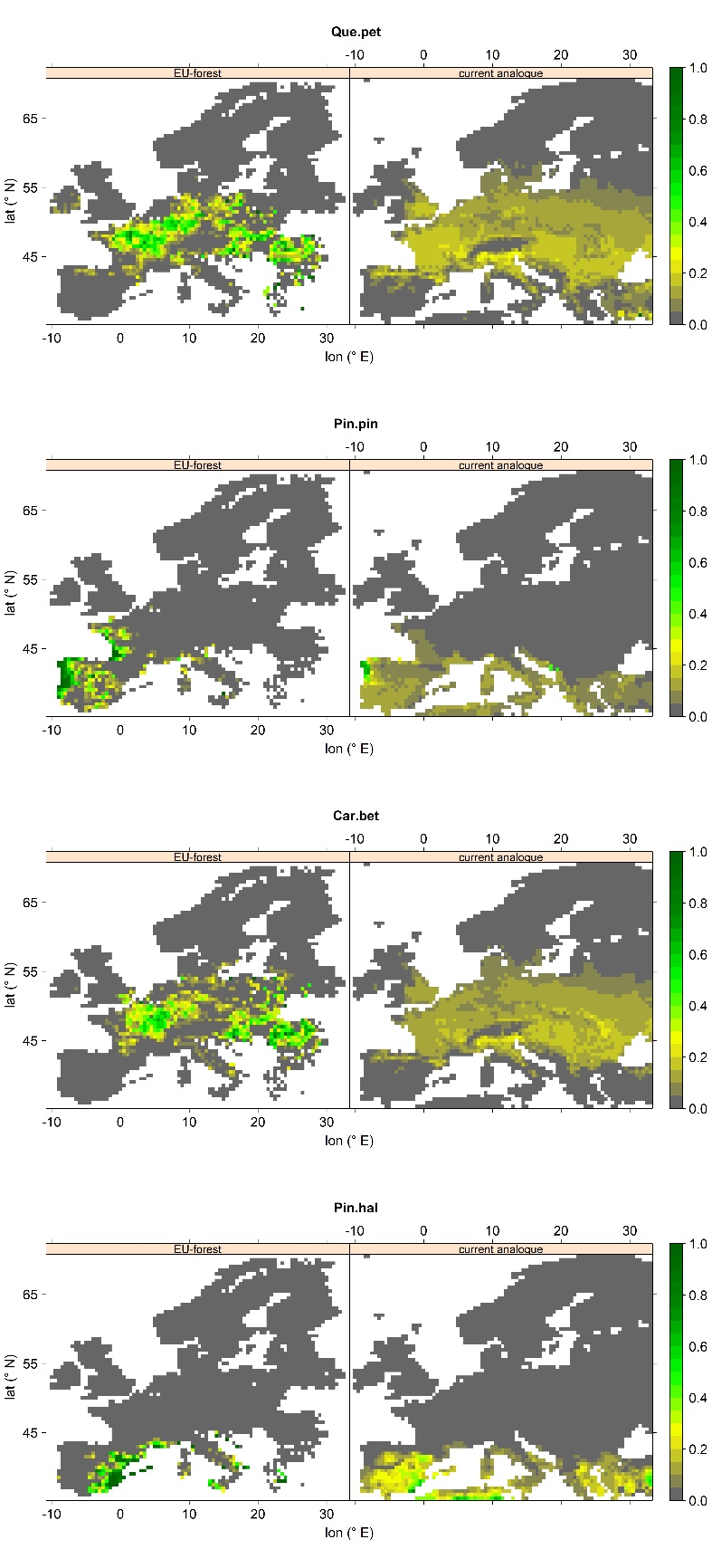


**Supplementary Figure S4.** Relative abundance of *Quercus petraea*, *Pinus pinaster*, *Carpinus betulus*, and *Pinus halapensis* according to EU-forest observations (left) and historic projections (right). Spearman’s rank correlation between EU-forest observations and historic projections are 0.59, 0.54, 0.62, and 0.47, respectively.


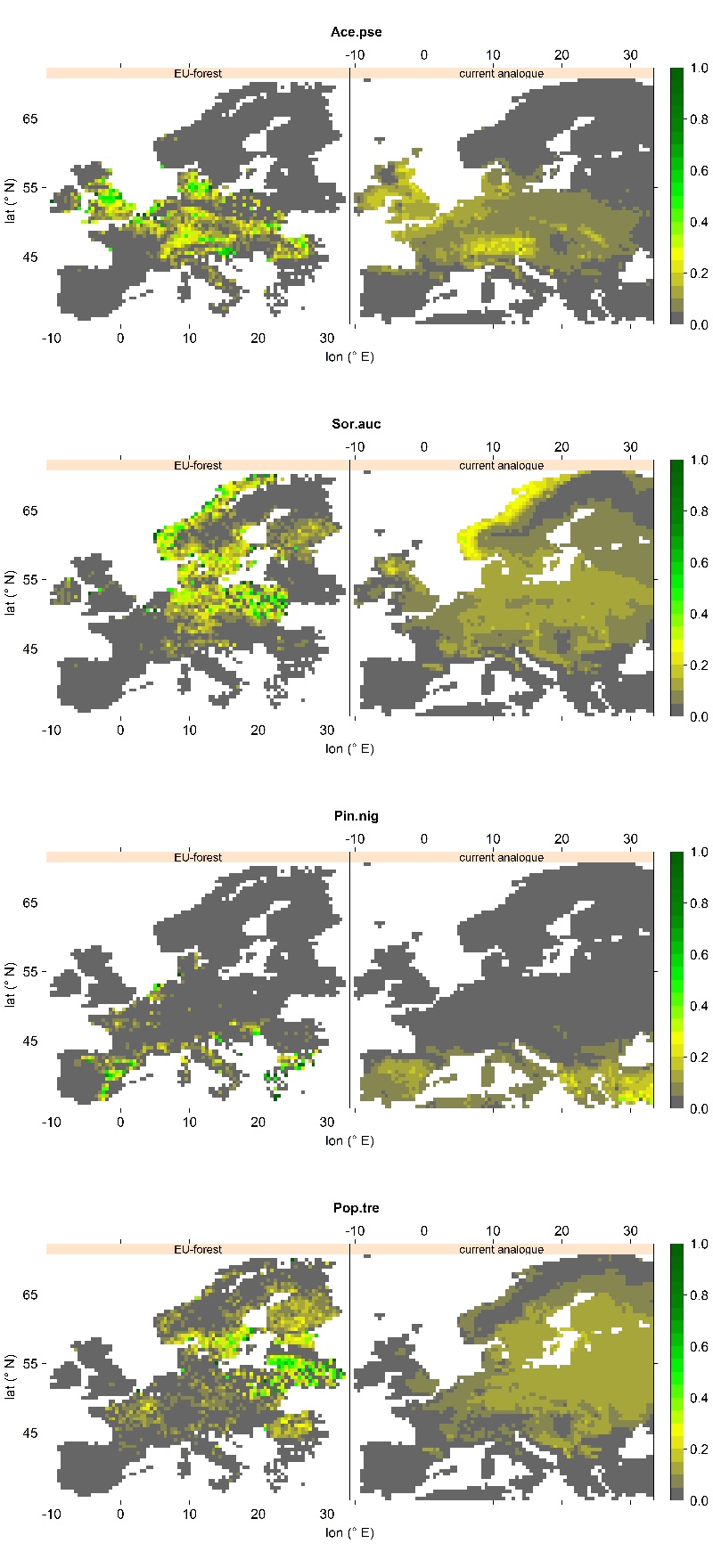


**Supplementary Figure S5.** Relative abundance of *Acer pseudoplatanus*, *Sorbus aucuparia*, *Pinus nigra*, and *Populus tremula* according to EU-forest observations (left) and historic projections (right). Spearman’s rank correlation between EU-forest observations and historic projections are 0.70, 0.55, 0.44, and 0.57, respectively.


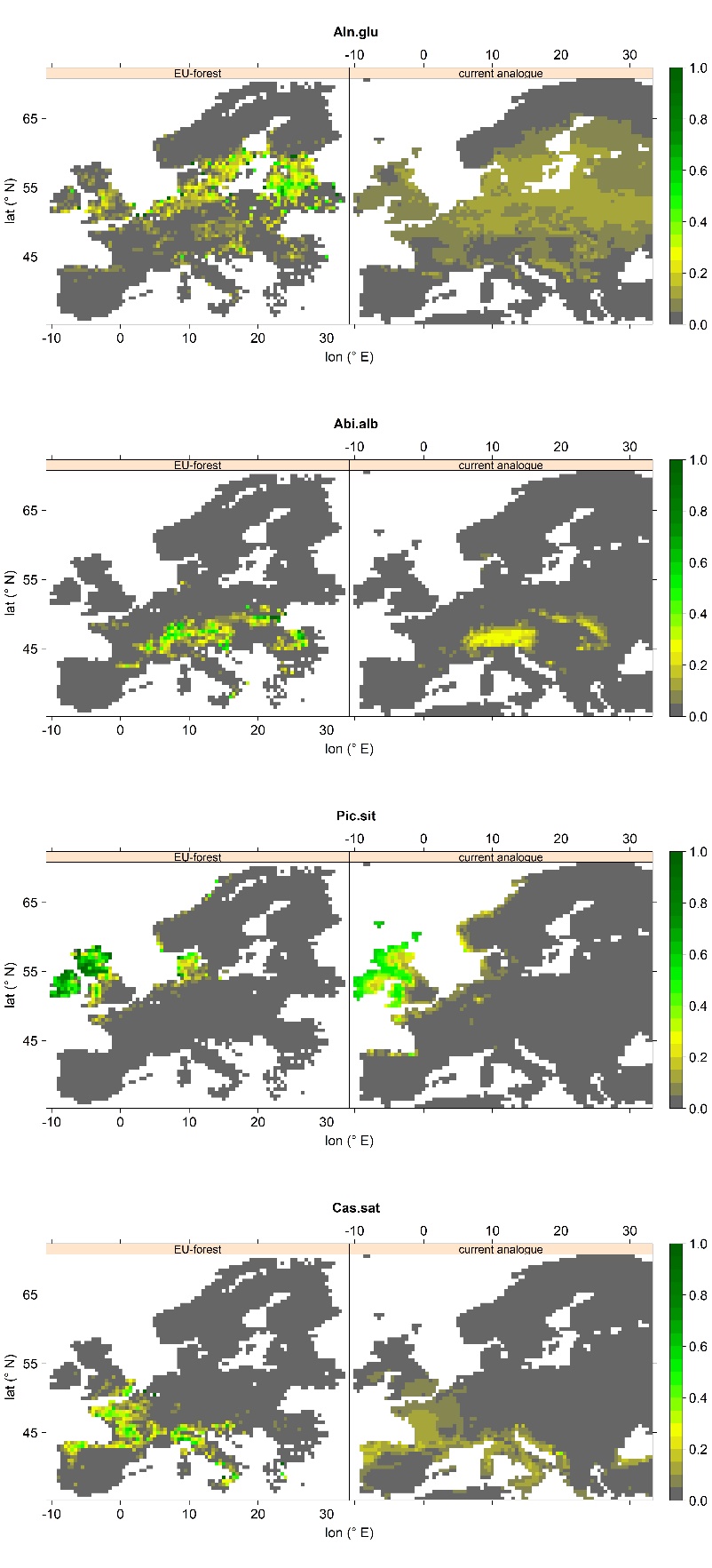


**Supplementary Figure S6.** Relative abundance of *Alnus glutinosa*, *Abies alba*, *Picea sitchensis*, and *Castanea sativa* according to EU-forest observations (left) and historic projections (right). Spearman’s rank correlation between EU-forest observations and historic projections are 0.51, 0.54, 0.53, and 0.56, respectively.


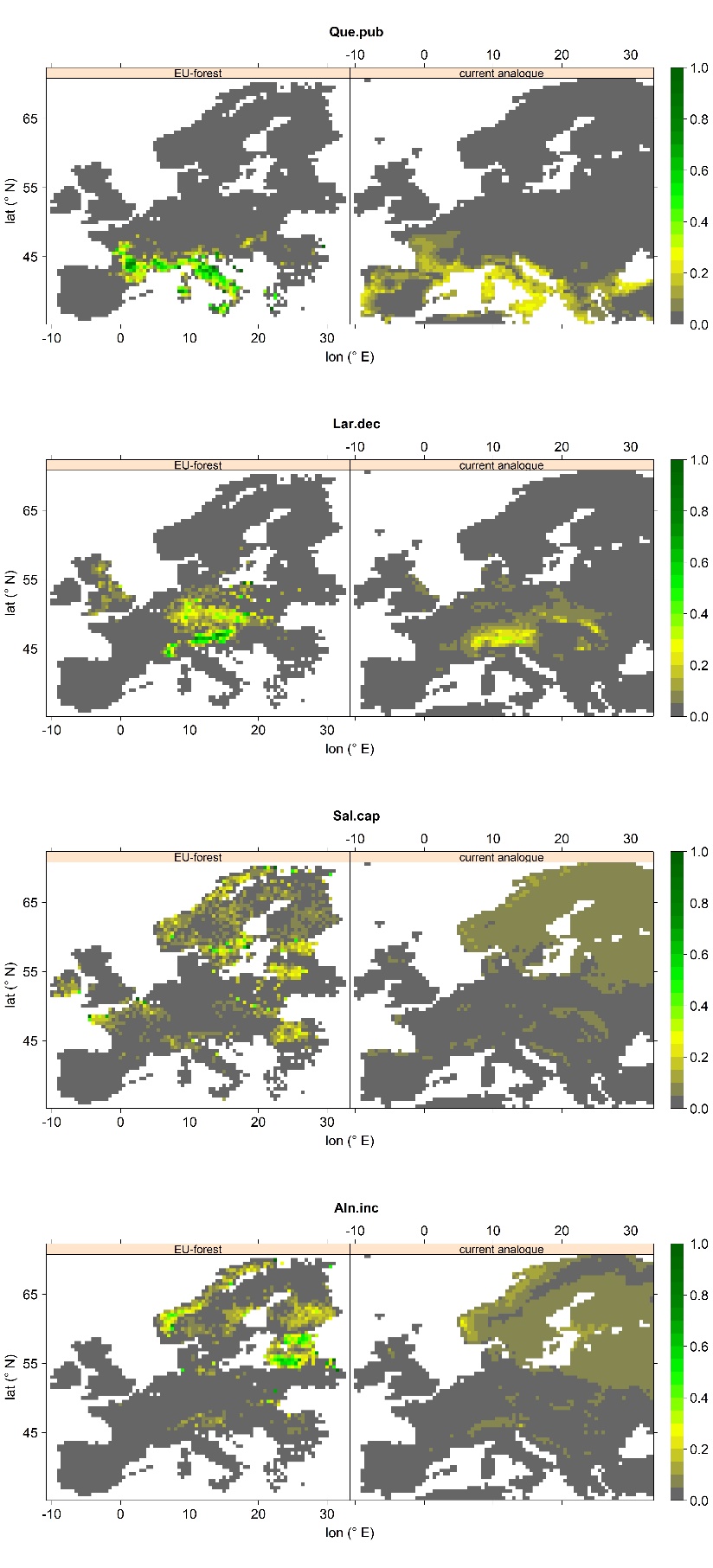


**Supplementary Figure S7.** Relative abundance of *Quercus pubescens*, *Larix decidua*, *Salix caprea*, and *Alnus incana* according to EU-forest observations (left) and historic projections (right). Spearman’s rank correlation between EU-forest observations and historic projections are 0.45, 0.54, 0.44, and 0.58, respectively.


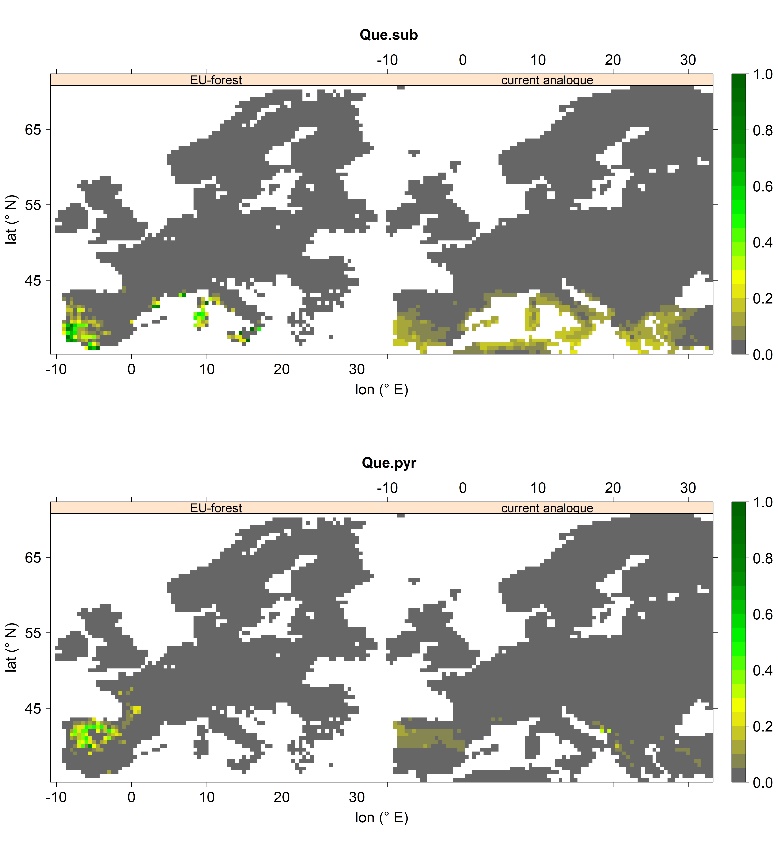


**Supplementary Figure S8.** Relative abundance of *Quercus suber* and *Quercus pyrenaica* according to EU-forest observations (left) and historic projections (right). Spearman’s rank correlation between EU-forest observations and historic projections are 0.46 and 0.43, respectively.


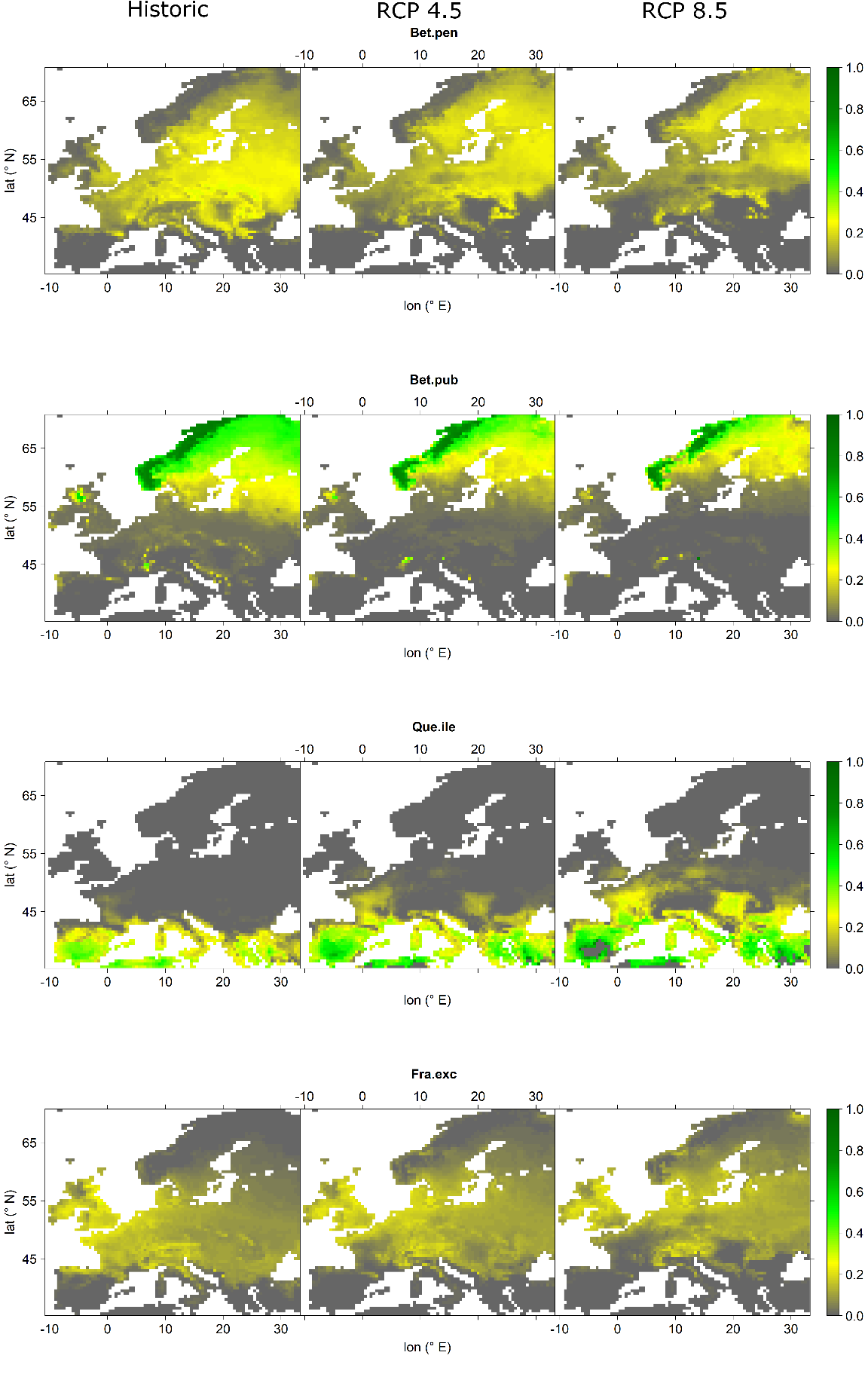


**Supplementary Figure S9.** Projected relative abundance probabilities for the tree species *Betula pendula*, *Betula pubescens*, *Quercus ilex*, and *Fraxinus excelsior* for current analogues (left panels), RCP 4.5 analogues (mid panels), and RCP 8.5 (right panels). Relative abundance probability increases from grey over yellow to green colors.


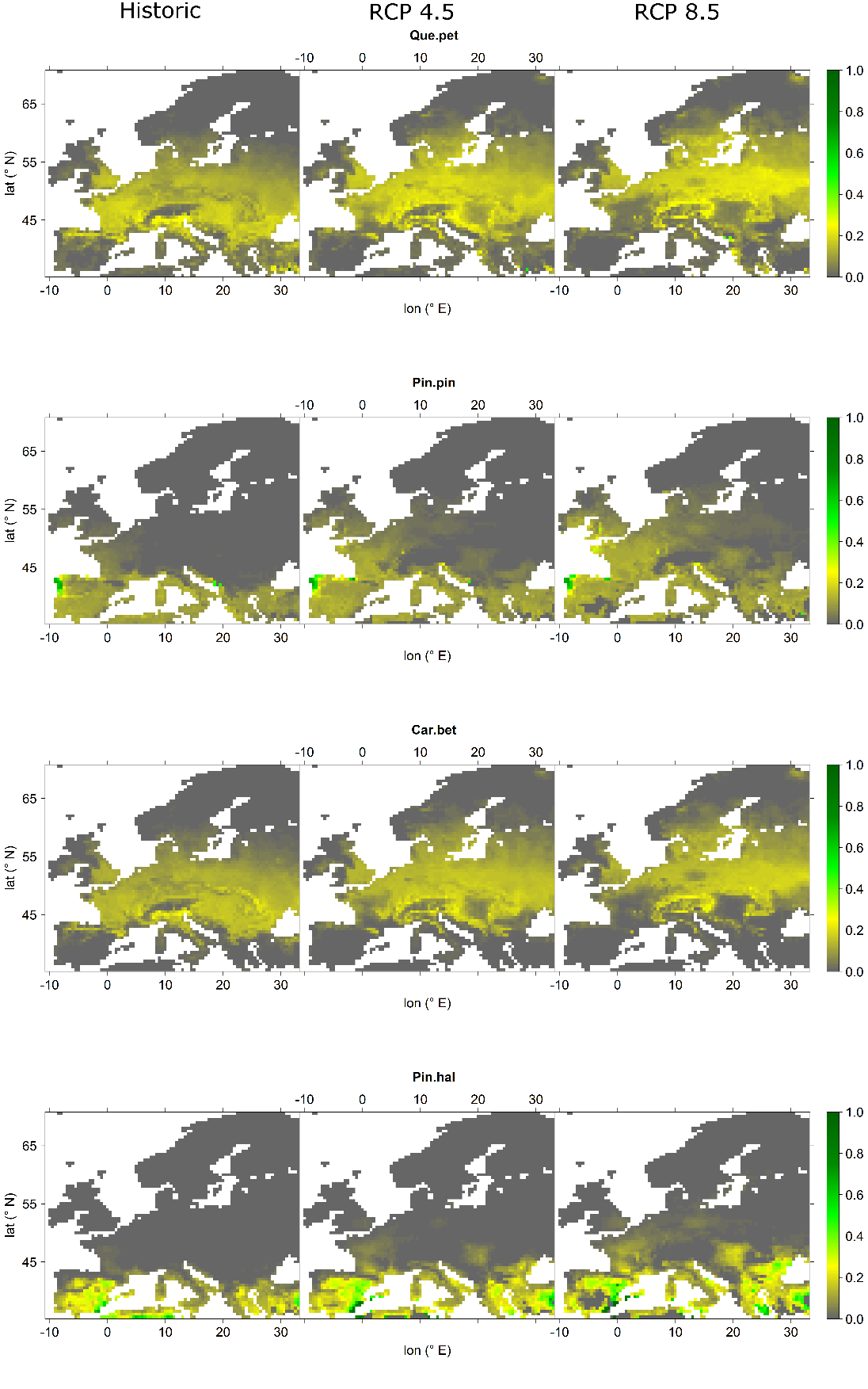


**Supplementary Figure S10.** Projected relative abundance probabilities for the tree species *Quercus petraea*, *Pinus pinaster*, *Carpinus betulus*, and *Pinus halapensis* for current analogues (left panels), RCP 4.5 analogues (mid panels), and RCP 8.5 (right panels). Relative abundance probability increases from grey over yellow to green colors.


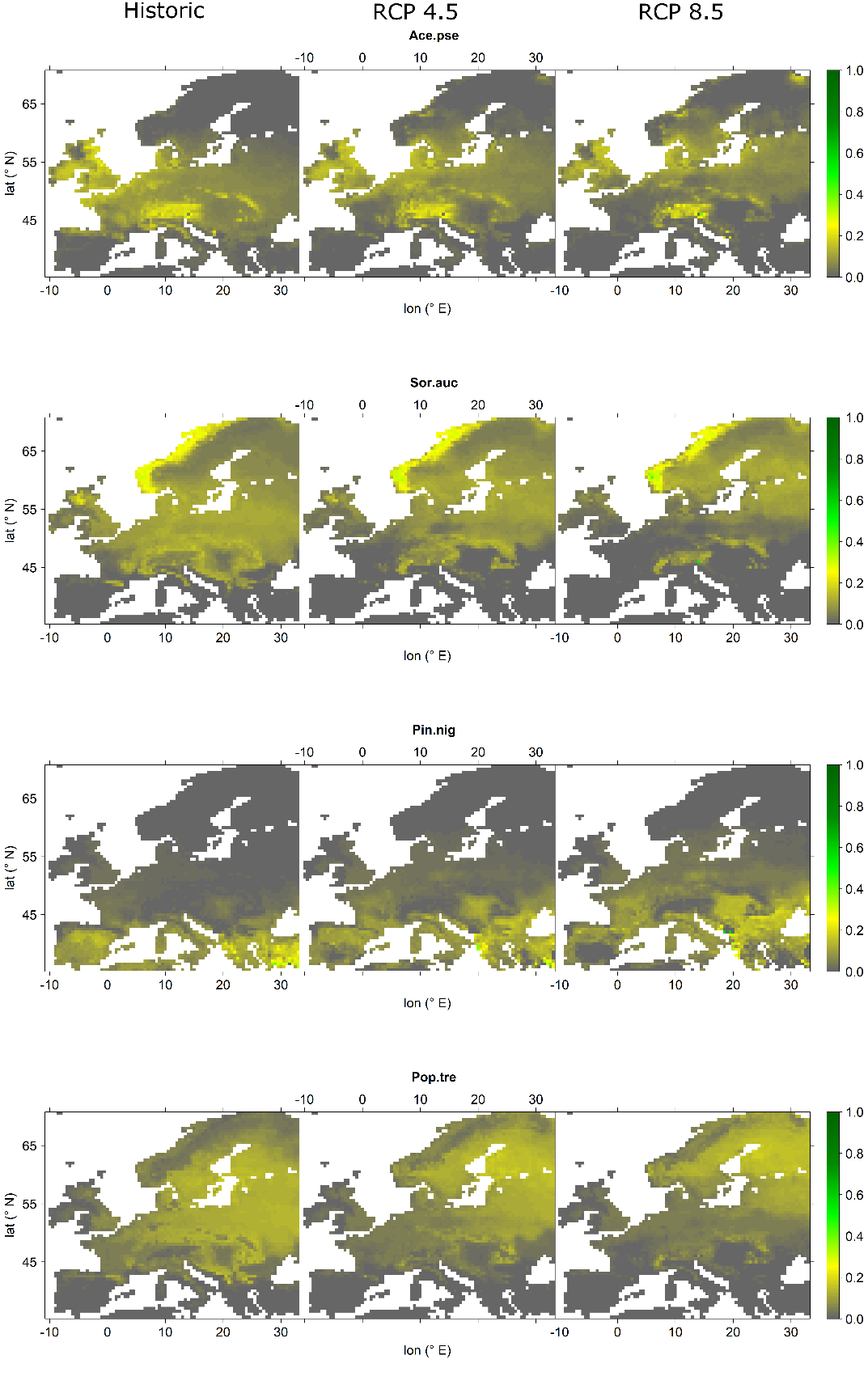


**Supplementary Figure S11.** Projected relative abundance probabilities for the tree species *Acer pseudoplatanus*, *Sorbus aucuparia*, *Pinus nigra*, and *Populus tremula* for current analogues (left panels), RCP 4.5 analogues (mid panels), and RCP 8.5 (right panels). Relative abundance probability increases from grey over yellow to green colors.


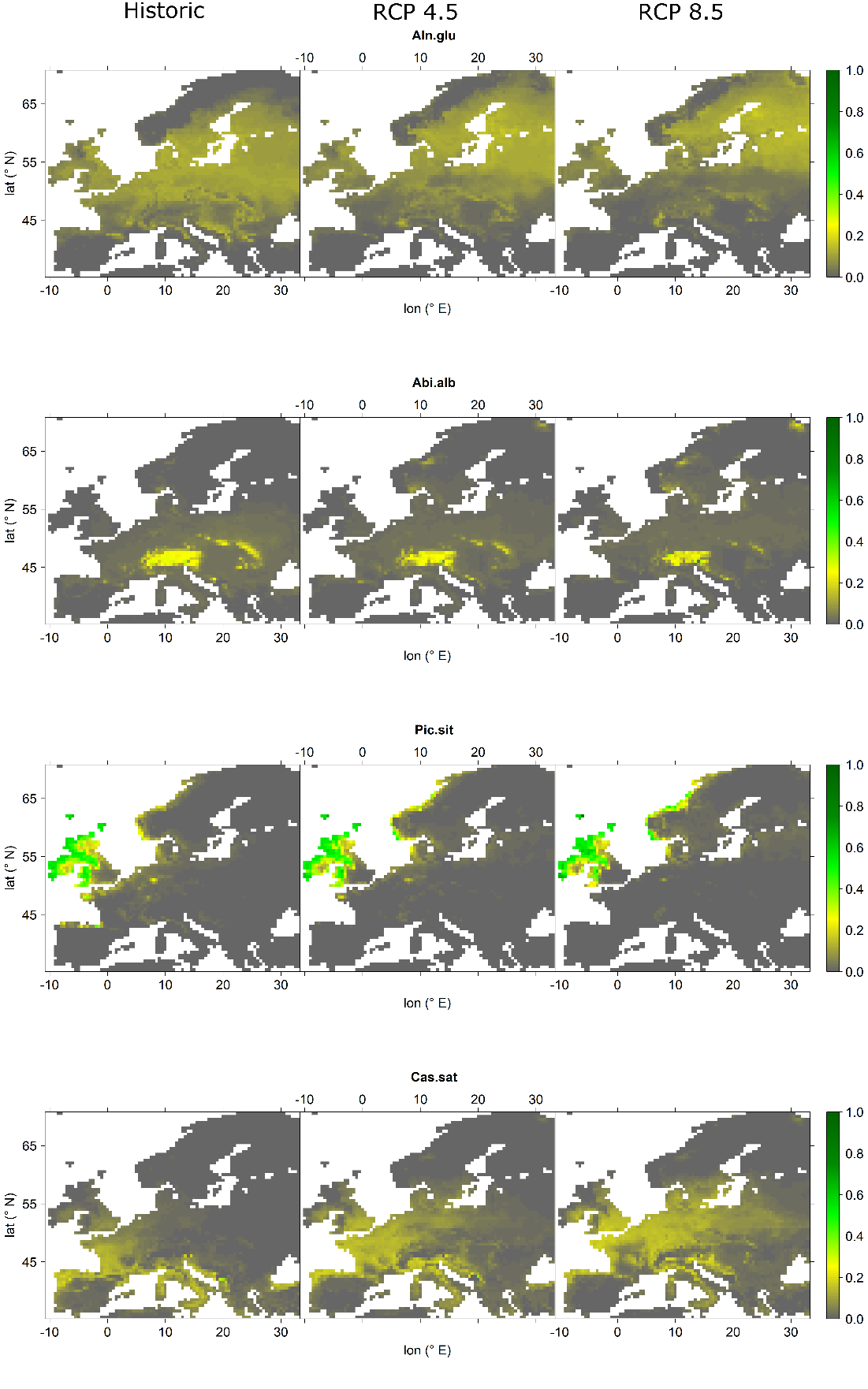


**Supplementary Figure S12.** Projected relative abundance probabilities for the tree species *Alnus glutinosa*, *Abies alba*, *Picea sitchensis*, and *Castanea sativa* for current analogues (left panels), RCP 4.5 analogues (mid panels), and RCP 8.5 (right panels). Relative abundance probability increases from grey over yellow to green colors.


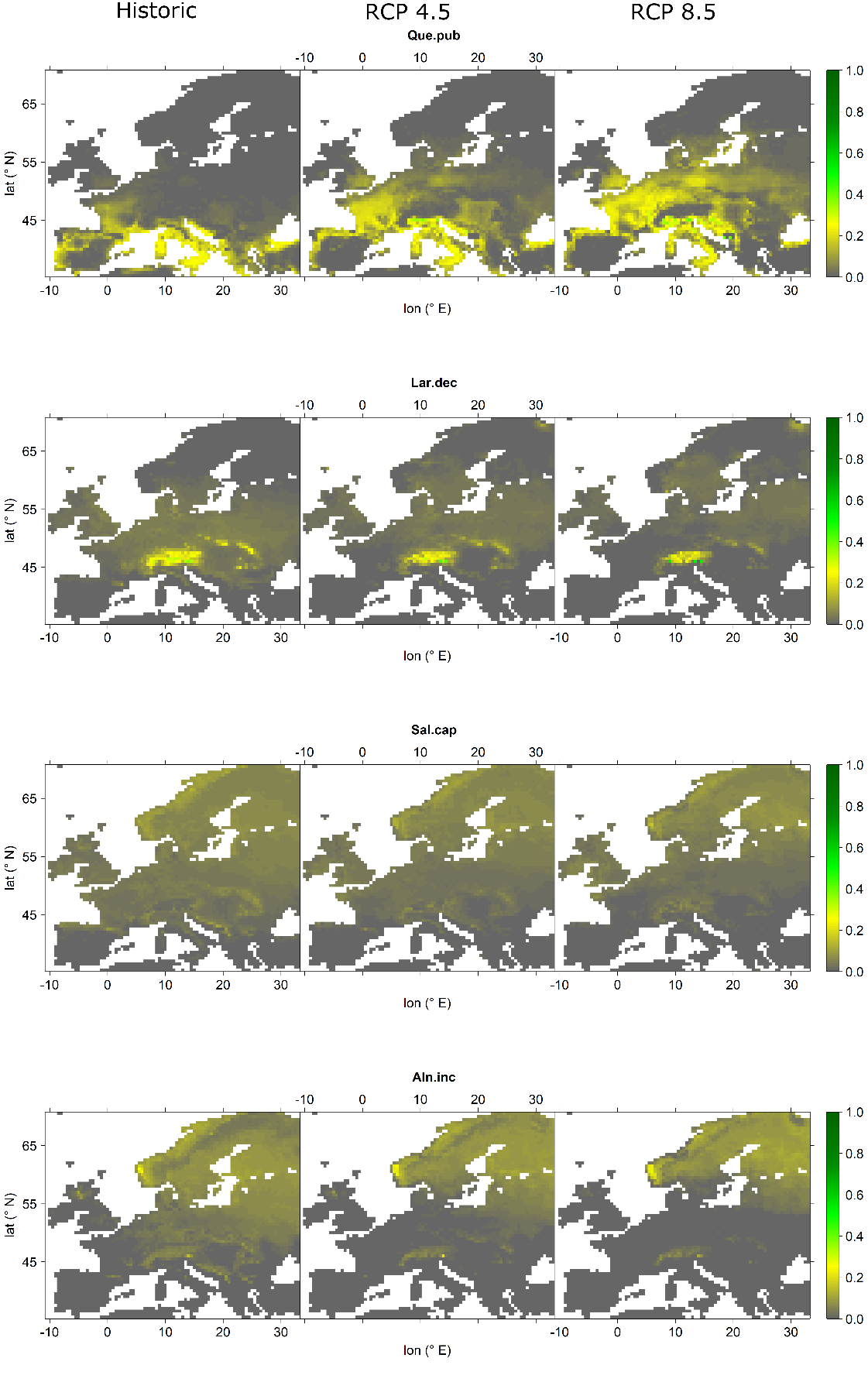


**Supplementary Figure S13.** Projected relative abundance probabilities for the tree species *Quercus pubescens*, *Larix decidua*, *Salix caprea*, and *Alnus incana* for current analogues (left panels), RCP 4.5 analogues (mid panels), and RCP 8.5 (right panels). Relative abundance probability increases from grey over yellow to green colors.


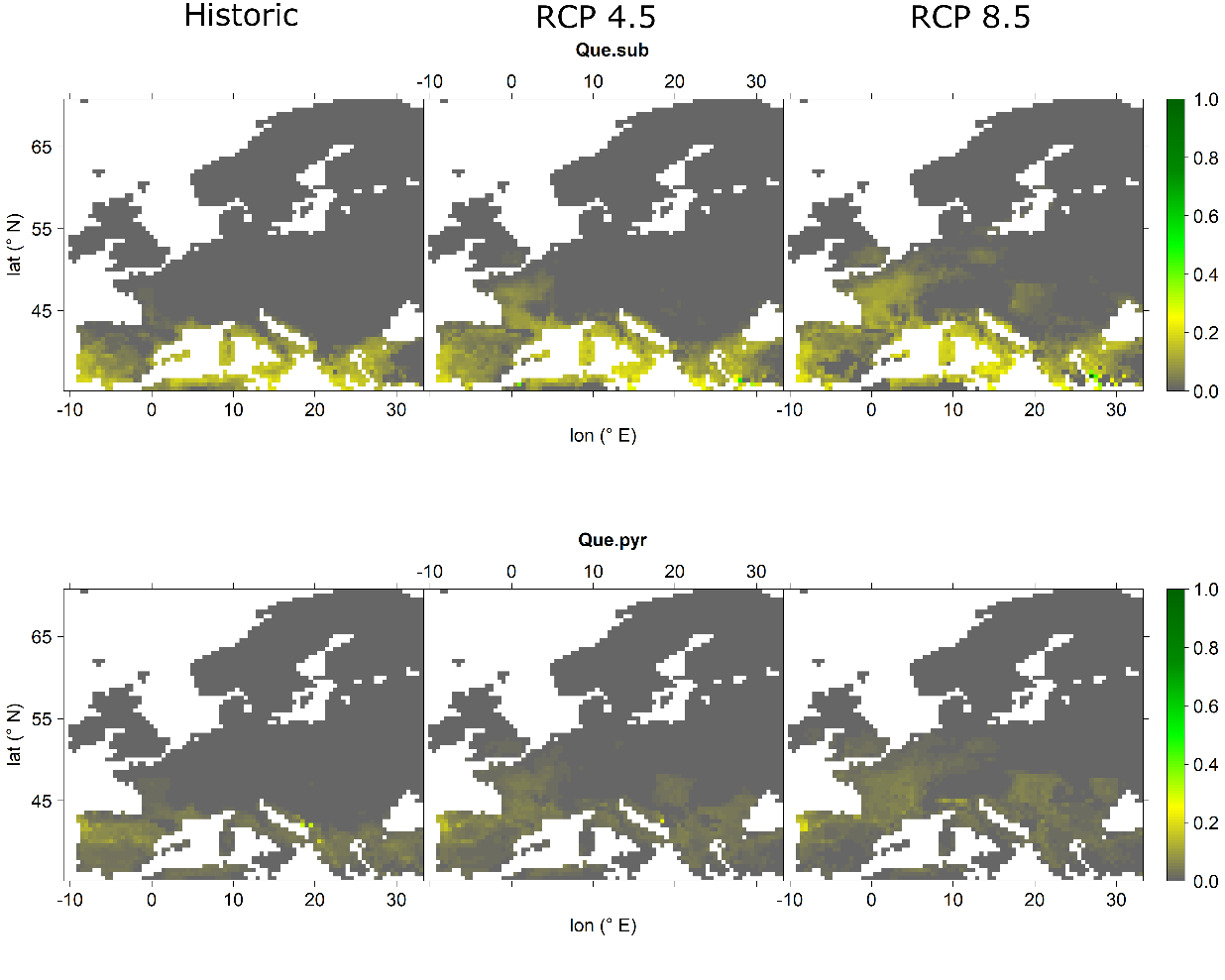


**Supplementary Figure S14.** Projected relative abundance probabilities for the tree species *Quercus suber* and *Quercus pyrenaica* for current analogues (left panels), RCP 4.5 analogues (mid panels), and RCP 8.5 (right panels). Relative abundance probability increases from grey over yellow to green colors.

## Supplementary Tables

**Supplementary Table S1.** Overview on the sixteen different CMIP5 climate models used for statistical downscaling of historic and future (RCP 4.5 and RCP 8.5) climate projections and their original spatial resolution.

| Model-acronym | Resolution (lat; lon) [°] |
| --- | --- |
| CMCC-CM | 0.7484; 0.7500 |
| CMCC-CMS | 3.7111; 3.7500 |
| FGOALS-g2 | 2.7906; 2.8125 |
| GFDL-ESM2G | 2.0225; 2.0000 |
| GFDL-ESM2M | 2.0225; 2.5000 |
| HadGEM2-AO | 1.2500; 1.8750 |
| HadGEM2-CC | 1.2500; 1.8750 |
| HadGEM2-ES | 1.2500; 1.8750 |
| INM-CM4 | 1.5000; 2.0000 |
| IPSL-CM5A-LR | 1.8947; 3.7500 |
| IPSL-CM5A-MR | 1.2676; 2.5000 |
| MIROC-ESM | 2.7906; 2.8125 |
| MIROC-ESM-CHEM | 2.7906; 2.8125 |
| MIROC5 | 1.4008; 1.4063 |
| MPI-ESM-LR | 1.8653; 1.8750 |
| MPI-ESM-MR | 1.8653; 1.8750 |

**Supplementary Table S2.** Variables used for the definition of climate analogues. T_min_ = mean monthly minimum temperature, T_mean_ = mean monthly mean temperature, T_max_ = mean monthly maximum temperature, P_sum_ = monthly precipitation sum. The term climatology refers to mean values over the climate normal periods 1961-1990 and 2061-2090.

| Parameter | Description | Calculation |
| --- | --- | --- |
| Growing season length | Length of the longest period where minimum daily temperatures were above 0° C | Interpolation of T_min_ to obtain daily values, climatology of length of longest period with consecutive days that fulfill T_min_ > 0 °C |
| Coldest month | Mean temperature of the coldest month | Climatology of T_mean_ for each month, extraction of the temperature of the coolest month |
| Warmest month | Mean temperature of the warmest month | Climatology of T_mean_ for each month, extraction of the temperature of the warmest month |
| Annual potential evapotranspiration | Annual sum of potential evapotranspiration | Climatology of annual sums of monthly PET which was derived from T_min_, T_max_, P_sum_, and the latitude using the Hargreaves function within the SPEI-package ^43–45^ |
| Wet days | Number of days with a positive water balance | monthly water balance was calculated as the difference between P_sum_ and monthly PET, monthly water balance was interpolated to daily values, number of days per year with positive daily water balance was extracted per year and used for the climatology |
| Driest month | Mean water balance (= Difference between P_sum_ and monthly PET) of the driest month | Climatology of monthly water balance (computed as for ‘wet days’), extraction of the driest month |
| Wettest month | Mean water balance (= Difference between P_sum_ and monthly PET) of the wettest month | Climatology of monthly water balance (computed as for ‘wet days’), extraction of the wettest month |
| Annual water balance | Sum of the annual water balance | Climatology of the sum of monthly water balances for each year |
| Temperature range | Range from coldest to warmest month | Warmest month minus coldest month |
| Annual variability of water balance | Inter-annual variation of monthly water balance | Calculation of the autocorrelation of the mean monthly water balance at a lag of 12 months (annual lag) |
| Seasonal variability of mean monthly temperature | Intra-annual variation of mean monthly temperature | Calculation of the autocorrelation of T_mean_ at a lag of 6 months (semi-annual lag) |

**Supplementary Table S3.** Overview on the 26 species from the EU-Forest dataset selected for further analysis and their respective proportional contribution to EU-Forest.

| Species | Proportional contribution [%] |
| --- | --- |
| *Pinus sylvestris* | 12.9 |
| *Picea abies* | 12.0 |
| *Fagus sylvatica* | 6.1 |
| *Quercus robur* | 5.3 |
| *Betula pubescens* | 4.5 |
| *Quercus ilex* | 3.9 |
| *Betula pendula* | 3.5 |
| *Fraxinus excelsior* | 3.0 |
| *Quercus petraea* | 2.9 |
| *Pinus pinaster* | 2.8 |
| *Carpinus betulus* | 2.2 |
| *Pinus halepensis* | 2.2 |
| *Acer pseudoplatanus* | 2.1 |
| *Sorcus aucuparia* | 1.8 |
| *Pinus nigra* | 1.8 |
| *Populus tremula* | 1.7 |
| *Alnus glutinosa* | 1.7 |
| *Abies alba* | 1.6 |
| *Picea sitchensis* | 1.6 |
| *Castanea sativa* | 1.5 |
| *Quercus pubescens* | 1.5 |
| *Larix decidua* | 1.4 |
| *Salix caprea* | 1.1 |
| *Alnus incana* | 1.1 |
| *Quercus suber* | 1.0 |
| *Quercus pyrenaica* | 1.0 |
